# Construction and validation of a prognostic model for lung adenocarcinoma based on necrotic cell death triggered by sodium overload-related coexpressed genes

**DOI:** 10.3389/fgene.2026.1763705

**Published:** 2026-03-02

**Authors:** Li Liang, Xiaoqi Wang, Huihui Liu, Ting Huang, Quan Zhu, Fangguo Lu, Chunjing Chen

**Affiliations:** 1 School of Acupuncture-Moxibustion, Tuina and Rehabilitation, Hunan University of Chinese Medicine, Changsha, China; 2 School of Medicine, Hunan University of Chinese Medicine, Changsha, China

**Keywords:** bioinformatics, cancer genomics, lung adenocarcinoma, necrotic cell death triggered bysodium overload (NECSO), prognostic model

## Abstract

**Introduction:**

The aim of this study was to explore the prognostic significance of necrotic cell death triggered by sodium overload (NECSO)-related genes in lung adenocarcinoma (LUAD) and construct a prognostic model with high predictive efficiency. The findings will enable a precise stratification of the prognostic risk of patients with LUAD. Analysis of the constructed prognostic model, immune cell infiltration, and tumor mutational burden (TMB) will facilitate the development of individualized precision medical protocols.

**Methods:**

Based on the LUAD data obtained from TCGA and GEO databases, a prognostic prediction model for LUAD containing 15 key genes (including arginyl aminopeptidase like 1 [RNPEPL1] and beta-1,3-N-acetylglucosaminyltransferase 3 [B3GNT3]) coexpressing with the key NECSO gene, TRPM4, was established.

**Results:**

Risk score was identified as an independent prognostic factor. High-risk patients had the following characteristics: high frequency of mutations in TP53 and TTN genes, high TMB, high number of immunosuppressive cells with impaired immune cell function, and abnormally active metabolism. Nomograms developed by integrating clinical features and risk scores displayed high predictability.

**Discussion:**

The findings provide new molecular markers and potential therapeutic targets for the prognosis of LUAD and lay a theoretical foundation for the development of therapeutic approaches targeting the NECSO mechanism in patients with LUAD.

## Introduction

Lung cancer is a malignant tumor with the highest morbidity and mortality rates worldwide, with approximately 2.2 million new patients diagnosed each year (Siegel et al., 2023). Non-small cell lung cancer (NSCLC) accounts for approximately 85% of all lung cancer cases, with lung adenocarcinoma (LUAD) being the most common subtype of NSCLC (Huang, 2011). Surgical resection remains the first choice for the treatment of LUAD. In recent years, molecular targeted therapy and immunotherapy have rapidly developed, bringing hope for the clinical treatment of a range of malignant tumors. However, the 5-year overall survival (OS) rate of patients with LUAD is still unsatisfactory because lung cancer is highly heterogeneous and difficult to diagnose in the early stages. Therefore, predictive prognostic models for patients with LUAD are urgently needed to identify new targets for the treatment of LUAD and improve the OS rate of patients.

Necrotic cell death triggered by sodium overload (NECSO) is a novel sodium-dependent cell death mechanism triggered by the aberrant activation of the transient receptor potential melastatin 4 (TRPM4) channel; this phenomenon leads to abnormal opening of the channels, initiating sodium inflow and membrane depolarization, resulting in the overload of Na^+^-K^+^ ATPases, leading to energy depletion and mitochondrial dysfunction, and ultimately, the characteristic necrotic cell death (Fu et al., 2025). In the field of tumor therapy, NECSO can induce sodium overload-associated necrosis in tumor cells through targeted activation of TRPM4, overcoming the resistance of traditional apoptotic pathways. The energy-depletion mechanism specifically targets tumor cells with abnormal metabolism and produces synergistic effects with existing drugs. This discovery opens new avenues for targeted tumor therapy. However, the specific mechanism underlying the role of NECSO in the occurrence and development of LUAD has not yet been discovered, and its significance in the treatment and prognosis of LUAD requires further elucidation. Therefore, in the current study, we constructed a NECSO-related LUAD prognostic model to predict the OS of patients with LUAD using the relevant public databases and analyzed immune cell infiltration and tumor mutational burden (TMB) based on this model.

## Methods

### Data sources and analytical tools

The data for this study were obtained from The Cancer Genome Atlas (TCGA) database (https://portal.gdc.cancer.gov/) (Cancer Genome Atlas Research Network et al., 2013; Liu et al., 2018; Tomczak et al., 2015), the gene expression database of the National Center for Biotechnology Information (NCBI; Gene Expression Omnibus, GEO; http://www.ncbi.nlm.nih.gov/geo) (Barrett et al., 2013; Clough and Barrett, 2016), and the literature on NECSO-related genes (ISSN 1552-4469\ISSN 1552-4450, online\print). The analysis tools included the R language (version 4.4.0) (Ihaka and Gentleman, 1996) and Perl (version 5.30.0) (Stajich et al., 2002), both of which are widely used in bioinformatics.

### Experimental methods

#### Data acquisition and organization

We downloaded LUAD RNA-seq and clinical data from the TCGA database. The downloaded data were cleaned using Perl. Subsequently, we acquired the expression profile data for 59 normal and 541 LUAD tissues. These data were used as the training set for constructing the prognostic models. Expression and clinical data were downloaded from the GEO database, and the filtering conditions were as follows: the number of tumor samples was greater than 30, and samples were required for each stage of LUAD. According to the filtering conditions, the GSE68465 data and platform file GPL96 were selected. The raw data were cleaned using Perl, and the cleaned data were used as the validation set.

#### Coexpression analysis and network diagrams

The cleaned TCGA RNA-seq data were screened to detect genes coexpressing with the NECSO gene TRPM4 using the “limma” (Ritchie et al., 2015) package of R, version 4.4.0; the filters corFilter = 0.3 and pvalueFilter = 0.001 were used to detect NECSO-related genes involved in LUAD. Network diagrams were visualized using the “reshape2” and “igraph” packages of R, version 4.4.0.

#### Gene ontology (GO) and Kyoto encyclopedia of genes and genomes (KEGG) enrichment analysis

The coexpressed genes were analyzed using the “DOSE” (Yu et al., 2015) and “clusterProfiler” (Yu et al., 2012) packages of R, version 4.4.0. Using the GO database, we analyzed the biological processes (BPs), molecular functions (MFs), and cellular components (CCs) regulated by the coexpressed genes. The KEGG database was used to evaluate the biological pathways associated with the coexpressed genes.

#### Extraction of expression data of coexpressed genes and merging with clinical data

The expression data of coexpressed genes were extracted from the cleaned TCGA RNA-seq and GEO expression data using the “limma” and “sva” (Leek et al., 2012) packages of R, version 4.4.0. The TCGA and GEO coexpression data were merged with clinical data.

#### Construction of prognostic model

The merged data were screened for prognosis-related genes using the “survival” and “survminer” packages of R, version 4.4.0, with a coxPfilter value = 0.05 and initially identified genes significantly associated with overall survival (OS). Using the “glmnet” (Engebretsen and Bohlin, 2019) and “survival” packages of R, version 4.4.0 to perform Least Absolute Shrinkage and Selection Operator (LASSO) regression analysis; the optimal penalty parameter was determined by selecting the point with the minimum cross-validation error, and finally, 15 NECSO-related genes significantly correlated with OS were screened out to establish the prognostic model formula. The corresponding regression coefficients used to calculate the risk score (RS) of each patient sample were as follows: risk score = coefficient (Gene 1) × expression (Gene 1) + coefficient (Gene 2) × expression (Gene 2) + … + expression (Gene n) × coefficient (Gene n). Here, “coefficient” refers to the regression coefficient obtained after performing LASSO regression, and “expression” refers to the expression of NECSO-related genes in each sample.

Using the model formula, the training and validation set RS values were calculated separately. The patients in the training and validation sets were divided into two groups, high- and low-risk, according to the median values of their RS. A patient was included in the high-risk group if the patient’s RS was greater than the median. Conversely, the patients whose RS values were lower than the median were included in the low-risk group. Survival analysis was conducted using the Kaplan-Meier method to determine whether the difference in survival between the two groups of patients was significant (results were considered statistically significant if p < 0.05 was obtained using the log-rank test).

#### Judgment of predictive capability of the model

To examine the predictive performance of the prognostic model, we performed receiver operating characteristic (ROC) analysis of the training set using the “timeROC” (Blanche et al., 2013) package of R, version 4.4.0. We constructed time-dependent ROC curves and computed the ROC of the RS model for the areas under the curve (AUCs) of the one-, three-, and 5-year survival rates. Subsequently, we evaluated the prediction accuracy based on the AUC values. We examined whether the prognostic model was associated with age, sex, and tumor stage by performing univariate and multivariate Cox regression analyses using the training set; the results were used to evaluate whether the prognostic model could be used as an independent prognostic factor for patients with LUAD. In addition, we constructed ROC curves to compare the effects of age, sex, stage, and RS on patient survival.

#### Construction and validation of nomogram

Using the “survival,” “regplot,” “rms,” and “survcomp” packages of R, version 4.4.0, column plots were constructed using data on sex, age, stage, and RS values to predict the OS rate of patients with LUAD after one, three, and 5 years of diagnosis. Calibration curves were plotted to illustrate the consistency between actual and predicted outcomes.

#### Gene set enrichment analysis (GSEA)

GSEA was performed using the TCGA RNA-seq and RS data using R, version 4.4.0, packages “limma,” “org.Hs.eg.db,” “DOSE,” “clusterProfiler,” and “enrichplot.” The signal transduction pathways that were differentially expressed in the low- and high-risk groups were screened.

#### Immune cell infiltration and correlation analysis

TCGA RNA-seq data were normalized using the “e1071,” “preprocessCore,” and “limma” packages; the abundance of 22 infiltrating immune cells in all samples in the TCGA training set was quantified using the CIBERSORT (Newman et al., 2015) algorithm. Subsequently, the findings were integrated with the RS values of the training set. A correlation heatmap was generated to visualize the correlations between immune cells and RS values. A vioplot was also used to compare the differences in the proportions of immune cells between the low- and high-risk groups of the training set (results were considered statistically significant if p < 0.05 was obtained using the log-rank test).

#### Tumor mutational burden (TMB) and survival analysis

TMB (Samstein et al., 2019) data were downloaded from the TCGA database and collated; the data for the high- and low-risk groups of the training set were analyzed using the “maftools” (Mayakonda et al., 2018) package of R, version 4.4.0, to calculate the TMB (mutations per million bases) for each patient. The mutation data were visualized using a waterfall chart. The TMBs of the high- and low-risk groups in the training set were compared using a vioplot. Survival analysis was performed using the Kaplan-Meier method to compare the survival differences between the groups with high and low TMB values. In addition, survival analysis was performed in conjunction with evaluation of RS values to compare the survival differences between the groups with high TMB + high RS, high TMB + low RS, low TMB + high RS, and low TMB + low RS; the results were considered statistically significant if p < 0.05 was obtained using the log-rank test.

### Statistical analysis

Statistical software in the R language, version 4.4.0, was used to process the data downloaded from the TCGA and GEO databases. Differences between the high- and low-risk groups were analyzed using vioplots. Survival analysis was conducted using the Kaplan-Meier method with the log-rank test. Perl, version 5.30.0, was used to integrate the expression profile data, clinical information downloaded from the TCGA and GEO databases, and TMB data. Differences were considered statistically significant at p < 0.05.

## Results

### Genes coexpressed in NECSO and LUAD and network diagrams

**FIGURE 1:**
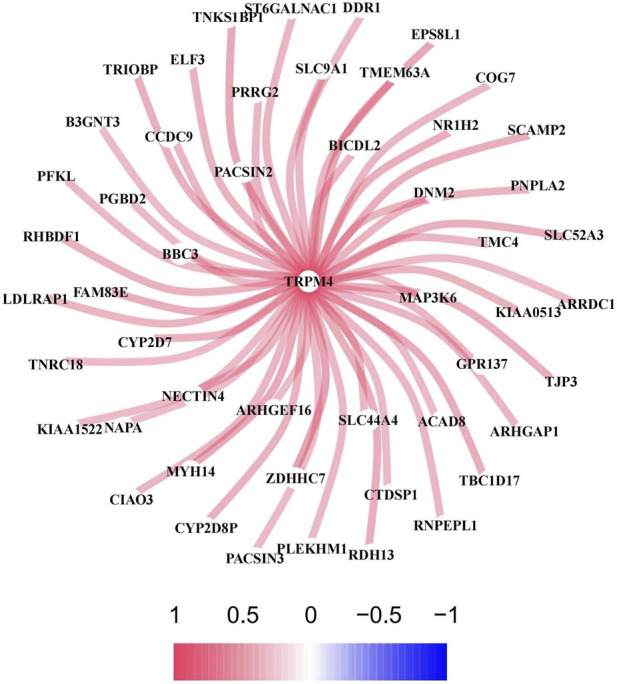
Co-expression network of NECSO gene with LUAD-related genes. TRPM4 serves as the central node, with the color gradient ranging from pink (correlation coefficient = 1) to blue (correlation coefficient = −1) indicating the strength of co-expression correlation between genes. Pink/red represents positive correlation (0∼1), blue represents negative correlation (−1∼0), and darker color denotes stronger correlation.

The 600 LUAD samples downloaded from the TCGA database were used as the training set, whereas the 443 LUAD samples downloaded from the GEO database were used as the validation set. Genes coexpressed with the NECSO gene TRPM4 in the training set were screened under the conditions of corFilter = 0.3 and p < 0.05. The analysis yielded 169 coexpressed genes, and the top 50 highly correlated genes were visualized (Figure 1).

### GO and KEGG analysis of NECSO-related genes

**FIGURE 2:**
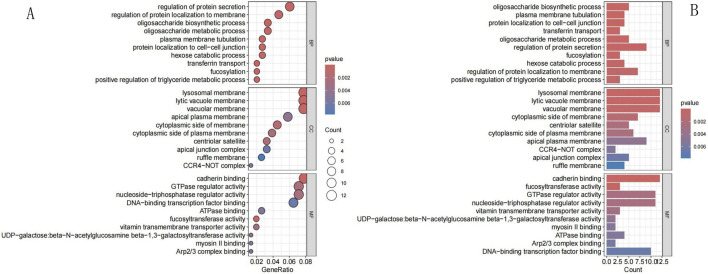
GO analysis. **(A)** GO Bubble chart. **(B)** GO Barplot. The left panel is a bubble chart and the right panel is a barplot. The x-axis of the bar chart represents the number of enriched genes (Count), while the x-axis of the dot plot denotes GeneRatio (the ratio of enriched genes to background genes). The y-axis shows the names of GO terms (including Biological Process, Cellular Component and Molecular Function) and KEGG pathways. Color intensity indicates the FDR/p-value, with darker color representing higher statistical significance. The size of dots in the right panel corresponds to the number of enriched genes (Count), with larger dots indicating more enriched genes.

**FIGURE 3:**
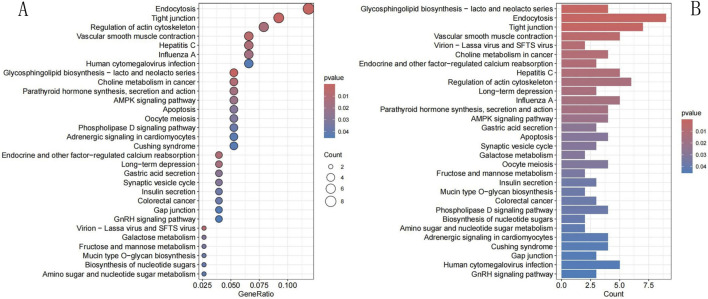
KEGG analysis. **(A)** KEGG Bubble chart. **(B)** KEGG Barplot. The left is a bubble chart and the right is a barplot. The x-axis of the bar chart represents the number of enriched genes (Count), and the x-axis of the dot plot represents GeneRatio (ratio of enriched genes to background genes). The y-axis indicates KEGG pathway names. Color intensity reflects FDR/p-value, with darker color representing higher statistical significance. The size of dots in the right plot corresponds to Count, and larger dots mean more enriched genes.

We analyzed 169 NECSO-related genes in GO and KEGG pathways. GO-based analysis (p < 0.05) showed that the genes were involved in BPs such as the regulation of protein secretion, protein localization to membrane, oligosaccharide biosynthetic process, and oligosaccharide metabolic process. Genes associated with CCs were mainly found in lysosomal, lytic vacuole, and vacuolar membranes. Genes associated with MFs mainly regulated cadherin binding, GTPase and nucleoside-triphosphatase activities, and DNA-binding transcription factor binding activity. Figure 2 shows the top 10 results for the BP, CC, and MF categories, ranked on the basis of the enrichment scores. The KEGG analysis (p < 0.05) revealed that the NECSO-related genes were mainly associated with endocytosis and regulation of tight junctions and actin cytoskeleton. Figure 3 shows the top 30 enriched pathways identified using KEGG analysis.

### Construction of a NECSO-related prognostic model for LUAD

**FIGURE 4:**
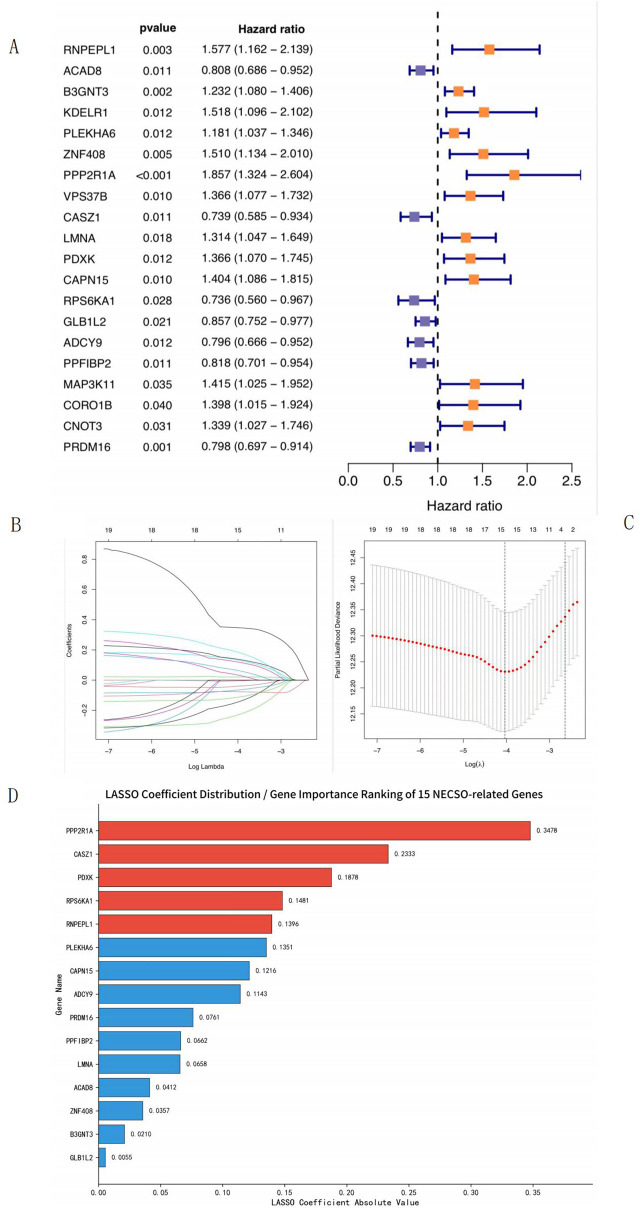
LASSO regression analysis. **(A)** Forest plot of hazard ratios for differentially expressed genes. **(B)** LASSO coefficient path plot. **(C)** LASSO deviance-λ relationship. **(D)** LASSO Coefficient Distribution/Gene Importance Ranking of 15 NECSO-related Genes. **(A)** This figure shows the hazard ratios (HR), 95% confidence intervals (95% CI), and corresponding p-values (pvalue) of 20 differentially expressed genes. Orange squares represent genes with a hazard ratio greater than 1 (high-risk genes), blue squares represent genes with a hazard ratio less than 1 (protective genes), and the length of the lines indicates the range of the 95% confidence interval. **(B)** It shows the change trend of feature coefficients with the logarithm of penalty parameter λ (Log Lambda): as Log Lambda decreases (λ increases), the coefficients of most features shrink gradually and some are compressed to 0, realizing feature selection. **(C)** It displays the relationship between partial likelihood deviance and Log(λ): the red dotted line represents the partial likelihood deviance at different λ values, with the lowest point corresponding to the optimal λ (marked by the dashed line), balancing model fitting effect and overfitting. The numbers at the top indicate the number of retained features under different λ values. **(D)** The horizontal axis represents gene names (ranked in descending order of contribution), and the vertical axis represents the absolute values of LASSO coefficients; the larger the value, the higher the contribution and weight of the gene to the lung adenocarcinoma prognostic model. Genes marked in red are core genes with high contribution, while those in blue are minor genes with low contribution, clearly reflecting the relative roles of each gene in the LASSO regression prognostic model.

Univariate Cox regression analysis (p < 0.05) of the training set revealed that 20 NECSO-related genes (13 upregulated and seven downregulated genes) were associated with the prognosis of patients with LUAD (Figure 4A). To further optimize the model and reduce the risk of overfitting, we performed LASSO regression analysis (Figures 4B,C); the results showed that 15 NECSO-related genes were associated with prognosis-related genes in patients with LUAD (Table 1) and the relative contribution of each gene to the final model (Figure 4D). Among these, arginyl aminopeptidase like 1 (RNPEPL1), beta-1,3-N-acetylglucosaminyltransferase 3 (B3GNT3), pleckstrin homology domain-containing family A member 6 (PLEKHA6), zinc finger protein 408 (ZNF408), protein phosphatase 2A (PPP2R1A), lamin A/C (LMNA), pyridoxal kinase (PDXK), and calpain 15 (CAPN15) were identified as risk factors for the prognosis of patients with LUAD, with hazard ratios (HRs) > 1. Moreover, acyl-CoA dehydrogenase 8 (ACAD8), castor zinc finger 1 (CASZ1), ribosomal protein S6 kinase A1 (RPS6KA1), galactosidase beta 1 like 2 (GLB1L2), adenylate cyclase 9 (ADCY9), protein tyrosine phosphatase receptor type F interacting protein-binding protein 2 (PPFIBP2), and PR domain-containing protein 16 (PRDM16) were protective prognostic factors in patients with LUAD with HR < 1.

**TABLE 1:** LASSO regression for prognostic analysis.

ID	Coeff	HR	HR.95L	HR.95H	p value
RNPEPL1	0.139615902901813	1.57666988148402	1.16194186141879	2.13942538583077	0.00345816450965638
ACAD8	−0.0411707222581777	0.808185437306731	0.685999022851651	0.952135031270913	0.0108826555592973
B3GNT3	0.0209777981372376	1.232214743659	1.07984812012707	1.40608030535992	0.00193081894822745
PLEKHA6	0.135145338896309	1.1814851599436	1.03738545790015	1.34560126376989	0.0119696306437253
ZNF408	0.0357273419058719	1.5097114561033	1.13410097753028	2.00972287816293	0.0047708244556977
PPP2R1A	0.347841344009843	1.85708653517014	1.32419998391376	2.60441809470286	0.000334086292021966
CASZ1	−0.233325937169438	0.739400937761452	0.585184044617346	0.934259489456882	0.0114145576449108
LMNA	0.0657758833033669	1.3141370469349	1.04715582263272	1.6491873900724	0.0183920588218012
PDXK	0.187754842630385	1.36640194934934	1.06975174082393	1.74531549324486	0.0124225477812048
CAPN15	0.12156559679314	1.40405505155393	1.08592503625562	1.81538367932983	0.00963155546551135
RPS6KA1	−0.148108019874895	0.735940371749953	0.560144339337829	0.966908335468884	0.0276935178132752
GLB1L2	−0.00553897468799589	0.857297082669048	0.752441085135074	0.976765227832987	0.020714638883554
ADCY9	−0.114294479491114	0.79637675719354	0.666253868248539	0.95191333157335	0.0123710240732109
PPFIBP2	−0.0661767043714598	0.817859419315966	0.700898084159598	0.954338504956647	0.0106639690876777
PRDM16	−0.0761425636466641	0.798185700987204	0.696881607884982	0.914216139516177	0.0011334520826692

Coeff reflects the direction and magnitude of the gene’s association with prognosis, while HR > 1 indicates the gene is a prognostic risk factor (elevated expression correlates with poor overall survival) and HR < 1 indicates a prognostic protective factor (elevated expression correlates with favorable overall survival). The 95% CI denotes the precision of the HR estimate; non-overlapping with 1 confirms statistical significance (defined as p value <0.05).

RS values were calculated for the samples in the training and validation sets. The RS for each sample was computed on the basis of coefficients obtained for each gene using the constructed model by LASSO regression; the calculation was as follows: (0.139615902901813 × Exp [RNPEPL1]) + (−0.0411707222581777 × Exp [ACAD8]) + (0.0209777981372376 × Exp [B3GNT3]) + (0.135145338896309 × Exp [PLEKHA6]) + (0.0357273419058719 × Exp [ZNF408]) + (0.347841344009843 × Exp [PPP2R1A]) + (−0.233325937169438 × Exp [CASZ1]) + (0.0657758833033669 × Exp [LMNA]) + (0.187754842630385 × Exp [PDXK]) + (0.12156559679314 × Exp [CAPN15]) + (−0.148108019874895 × Exp [RPS6KA1]) + (−0.00553897468799589 × Exp [GLB1L2]) + (−0.114294479491114 × Exp [ADCY9]) + (−0.0661767043714598 × Exp [PPFIBP2]) + (−0.0761425636466641 × Exp [PRDM16]), where “Exp” refers to the expression levels of prognosis-related genes in each sample.

### Preliminary correlation analysis between target genes and clinical risk factors

**FIGURE 5:**
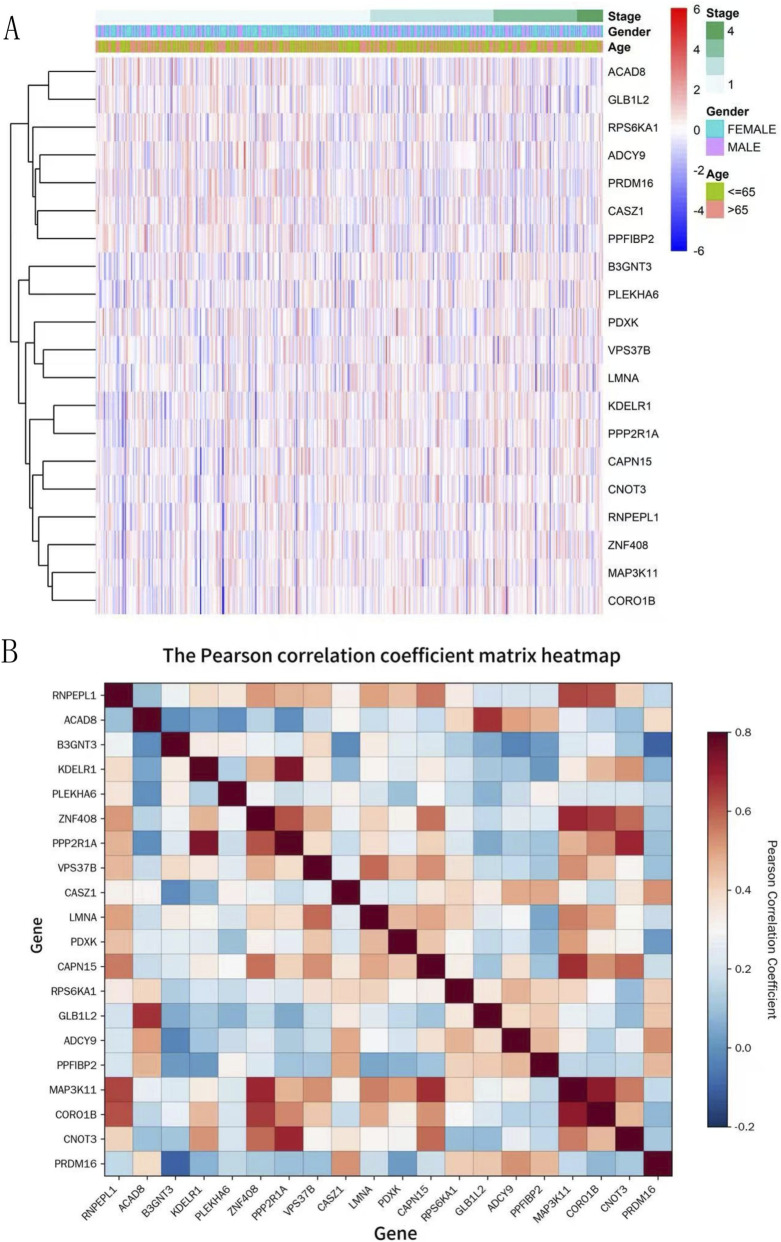
Preliminary correlation analysis between target genes and clinical risk factors. **(A)** Expression clustering heatmap of differentially expressed genes. **(B)** The Pearson correlation coefficient matrix heatmap. **(A)** Rows represent genes and columns represent samples; colors from red to blue indicate gene expression levels from high to low. The annotation bars at the top show the clinical stage, gender, and age information of the samples, respectively. **(B)** This figure shows the Pearson correlation coefficients among 20 target genes, with colors from red to blue indicating correlations from positive to negative. The color bar on the right shows the specific range of correlation coefficients.

The heatmap (Figure 5A) of gene expression and clinical features showed that genes such as RNPEPL1 and PPP2R1A exhibited significant expression differences in patients with high-stage (Stage 4) tumors, suggesting a close association with tumor progression risk. Genes including CAPN15 and PDXK displayed distinct expression patterns between patients aged ≥65 and <65 years, indicating potential links to age-related risks. In contrast, the impact of gender on gene expression was relatively weak, with only a few genes (such as GLB1L2) showing mild gender-specific differences.

The Pearson correlation coefficient matrix heatmap (Figure 5B) identified strong positive correlations (r > 0.6) between ZNF408 and PPP2R1A, as well as between MAP3K11 and CORO1B, indicating potential synergistic effects in risk-related pathways. A continuous positive correlation cluster (CASZ1-LMNA-PDXK-CAPN15-RPS6KA1) was also observed, suggesting this module may represent a core risk pathway for tumor progression. Additionally, PRDM16 showed significant negative correlations (r < −0.2) with ZNF408 and PPP2R1A, implying antagonistic roles in distinct risk regulatory mechanisms. Notably, genes such as ACAD8 and B3GNT3 exhibited weak associations with other genes and clinical features, suggesting they may act as independent prognostic risk factors.

### Validation of a NECSO-related prognostic model for LUAD

**FIGURE 6:**
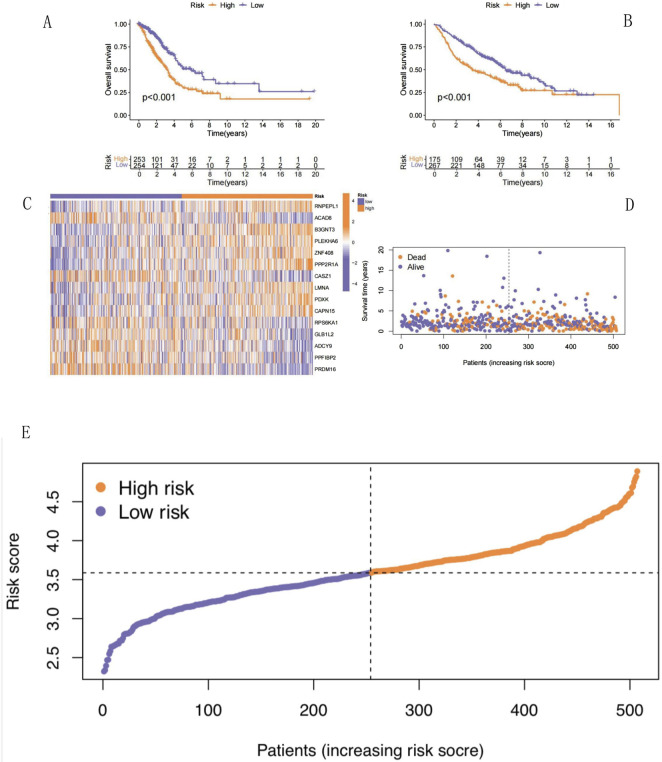
Survival analysis. **(A)** TCGA survival plot. **(B)** GEO survival plot. **(C)** The heatmap showing differential expression of NECSO-related genes. **(D)** Risk score analysis scatter plot. **(F)** The patient risk score ranking plot. **(A)** It shows the overall survival difference stratified by risk score. There is a significant difference in survival prognosis between the high-risk group (orange curve) and the low-risk group (blue curve) (p < 0.001). The sample size distribution of risk groups over time is attached below, intuitively presenting the sample size changes of risk groups at different time points. **(B)** In the independent validation cohort, the overall survival difference between the high-risk group (orange curve) and the low-risk group (blue curve) is also statistically significant (p < 0.001), further verifying the prognostic value of the risk score. The sample size distribution below helps illustrate the stability of the grouping. **(C)** It shows the expression profile differences of 15 NECSO-related genes (RNPEPL1, ACAD8, B3GNT3, etc.) between the high-risk group (marked in blue) and the low-risk group (marked in orange). The color gradient reflects the relative changes in gene expression levels, clearly presenting the correlation between risk grouping and gene expression patterns. **(D)** The abscissa is the increasing order of patients’ risk scores, and the ordinate is the survival status (death is orange, survival is blue). The dotted line is the risk cutoff point, intuitively showing the correlation between risk score and survival outcome. The proportion of deaths in patients with high-risk scores is significantly higher. **(E)** Samples are sorted by risk score from low to high, with the dashed line representing the cutoff value for high- and low-risk grouping. The purple curve represents the low-risk group and the orange curve represents the high-risk group, visually demonstrating the continuous change in risk scores and the grouping boundary.

After acquiring the RS values of the training and validation sets, the patients in the training and validation sets were divided into high- and low-risk groups according to the median RS obtained using the Kaplan–Meier survival analysis (Figures 6A,B). Regarding the log-rank test, p-values <0.001 for the training and validation sets indicated that the survival rates of the high- and low-risk groups were significantly different. In both the training and validation sets, as the RS increased, the survival time of patients decreased, and mortality risk of patients increased. We analyzed the differences in the expression levels of NECSO-related genes between the high- and low-risk groups in the training set using heatmap (Figure 6C) and scatterplot for survival analysis (Figure 6D). Figure 6D shows that the higher the RS in the scatterplot, the lower the number of surviving patients and the higher the mortality rate. Thus, the NECSO-related prognostic model constructed using the training set data had a strong predictive ability for the prognosis of patients with LUAD. The distribution characteristics were derived from the patient risk score ranking plot (Figure 6E): the risk scores of all patients showed a continuous increasing trend, ranging from approximately 2.5–4.8, and presented an overall right-skewed distribution pattern—most patients had scores concentrated in the low-to-moderate range of 2.5–3.5, while a small number of patients had scores reaching above 4.8. Taking 3.5 as the optimal cutoff value (screened by the log-rank test maximization method with cross-validation), patients were divided into high- and low-risk groups, with approximately 50% of patients assigned to the high-risk group.

**FIGURE 7:**
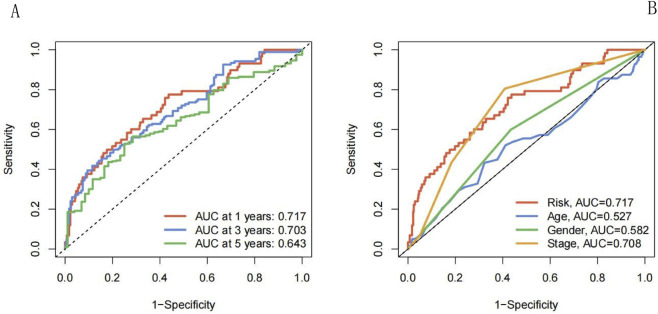
ROC curve. **(A)** Five year survival rate ROC curve. **(B)** ROC curve of clinical indicators. **(A)** It evaluates the predictive efficacy of the risk score for 1-year, 3-year, and 5-year survival rates. The AUCs are 0.717, 0.703, and 0.643, respectively, indicating that the risk score has a moderate to high predictive value for long-term survival. **(B)** It compares the predictive efficacy of the risk score with age, gender, and stage. The AUC of the risk score is 0.717, which is better than age (0.527) and gender (0.582), and slightly lower than stage (0.708), indicating that the risk score is an independent and effective prognostic predictor.

In addition, ROC curves were constructed based on the RS values of the training set to assess the predictive accuracy of the NECSO-related LUAD prognostic model. As shown in Figure 7A, the AUC values for one, three, and 5 years were 0.717, 0.703, and 0.643, respectively, in the training set. These outcomes suggested that the model predicted the survival of patients with LUAD with high accuracy and showed a good predictive performance. We also compared the effects of age, sex, stage, and risk index on patient survival separately; the results suggested (Figure 7B) that the risk index (AUC = 0.717) had a higher predictive value for prognosis than that of age (AUC = 0.527), sex (AUC = 0.582), and stage (AUC = 0.708).

**FIGURE 8:**
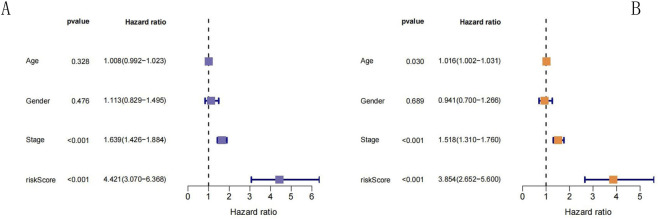
Cox regression analysis. **(A)** Univariate Cox regression analysis. **(B)** Multivariate Cox regression analysis. **(A)** Univariate Cox regression analysis: The forest plot shows the hazard ratio (HR) and statistical significance of age, gender, stage, and risk score. The HR of the risk score is 4.421 (p < 0.001), and the HR of stage is 1.639 (p < 0.001), suggesting that both are independent prognostic risk factors, while age and gender have no significant prognostic value. **(B)** Multivariate Cox regression analysis: After adjusting for age, gender, and stage, the HR of the risk score is still as high as 3.854 (p < 0.001), and the HR of stage is 1.518 (p < 0.001), further confirming that the risk score and stage are prognostic determinants independent of clinical indicators.

To evaluate the independence of predictions of the prognostic model, univariate and multivariate Cox regression analyses were performed for 495 patients with LUAD in the training group whose clinical history information was complete (including sex, age, and tumor stage). The results of the univariate Cox regression analysis showed (Figure 8A) that the RS [HR = 4.421; 95% confidence interval (CI), 3.07–6.368; p < 0.001] was the influencing factor that significantly affected the survival of patients with LUAD. After integration of the correlations between multiple clinical factors, the outcomes of the multivariate Cox regression analysis indicated (Figure 8B) that the RS (HR = 3.854; 95% CI, 2.652–5.6; p < 0.001) was significantly associated with the survival of patients with LUAD. Thus, both univariate and multivariate Cox regression analyses revealed that the RS was significantly associated with OS in patients with LUAD, suggesting that the RS can be used as a prognostic factor independent of sex, age, and tumor stage.

### Construction and application of a nomogram

**FIGURE 9:**
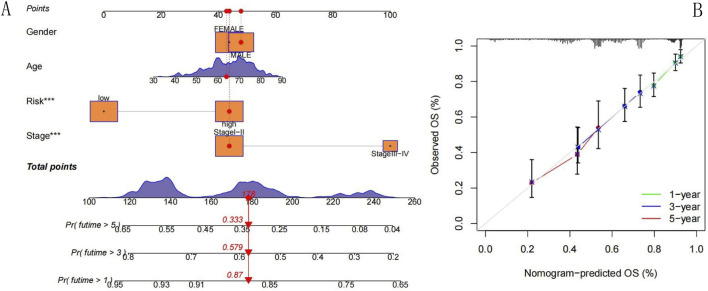
Nomogram. **(A)** Nomogram. **(B)** Calibration curve. **(A)** Nomogram: This nomogram integrates clinical factors (gender, age, risk group, tumor stage) to predict the probability of overall survival (OS) at 1, 3, and 5 years. Each factor is assigned points based on its prognostic weight. To use the nomogram, sum the points from each factor. Then, draw a vertical line down to the survival probability scales to read the predicted 1-, 3-, and 5-year OS probabilities. **(B)** Calibration curve: This curve assesses the agreement between the nomogram-predicted OS probability and the observed actual OS. The x-axis represents the nomogram-predicted OS percentage, and the y-axis represents the observed OS percentage. The green, blue, and red lines correspond to 1-year, 3-year, and 5-year OS, respectively. The closer the curves are to the diagonal reference line, the better the nomogram’s predictive accuracy. Error bars indicate the variability of predictions, showing that the nomogram has good calibration for short- and long-term OS, with minimal deviation between predicted and observed outcomes.

To predict the one-, three-, and five-year survival rates of patients with LUAD, we constructed a nomogram based on the prognostic model (Figure 9A), which combined information on age, tumor stage, sex, and RS. Using the nomogram, the survival probabilities of patients with LUAD after one, three, and five years of diagnosis were determined by drawing a vertical line from the total score axis of the outcome axis. We also plotted calibration curves (Figure 9B), and the three lines essentially coincided with the ideal line, highlighting the agreement between the actual and predicted outcomes. Therefore, this method can be clinically used to predict the survival of patients with LUAD.

### GSEA

**FIGURE 10:**
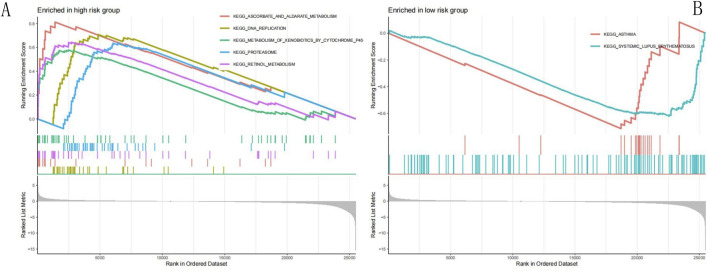
GSEA. **(A)** GSEA in high-risk group. **(B)** GSEA in low-risk group. **(A)** GSEA in high-risk group: This panel displays the gene set enrichment analysis (GSEA) results for the high-risk group, focusing on KEGG pathways. The running enrichment score (RES) curves (colored lines) illustrate the cumulative enrichment of each pathway as genes are ranked by their association with the high-risk phenotype. Five pathways are significantly enriched: KEGG_ASCORBATE_AND_ALDARATE_METABOLISM (red), KEGG_DNA_REPLICATION (yellow), KEGG_METABOLISM_OF_XENOBIOTICS_BY_CYTOCHROME_P45 (green), KEGG_PROTEASOME (blue), and KEGG_RETINOL_METABOLISM (purple). The peaks of these curves indicate strong enrichment, and the vertical bars below mark the positions of genes within each pathway in the ranked list. The ranked list metric (gray curve at the bottom) reflects the correlation of genes with the high-risk phenotype. These pathways suggest that high-risk patients exhibit enhanced activity in DNA replication, proteasomal degradation, and metabolic processes, potentially driving aggressive tumor behavior. **(B)** GSEA in low-risk group: This panel presents GSEA results for the low-risk group, also focusing on KEGG pathways. Two pathways are enriched:KEGG_ASTHMA (red) and KEGG_SYSTEMIC_LUPUS_ERYTHEMATOSUS (teal). The RES curves show negative enrichment scores, indicating these pathways are associated with the low-risk phenotype. The vertical bars below mark the positions of genes in these pathways within the ranked list. The ranked list metric (gray curve) reflects genes correlated with the low-risk phenotype. Enrichment of immune-related pathways suggests that low-risk patients may have distinct immune regulation, contributing to better prognosis.

We performed GSEA using the low- and high-risk groups of the training set of the NECSO-related LUAD prognostic model to investigate the potential molecular mechanisms associated with LUAD; 36 KEGG pathways were identified. The top five pathways in the high-risk group and the top two pathways in the low-risk group are summarized in Table 2 and Figures 10A,B (Note: ES, enrichment score; NES, normalized enrichment score).

**TABLE 2:** GSEA of KEGG pathways between high-risk and low-risk groups.

Enriched pathways	Size	Es	NES	p value	p. adjust	q value
High risk group						
KEGG_DNA_REPLICATION	34	0.708340875	2.272118142	4.40E-06	0.000620406	0.00048632
KEGG_PROTEASOME	42	0.635871141	2.106421768	2.08E-05	0.000774974	0.000607483
KEGG_RETINOL_METABOLISM	43	0.643673729	2.148424351	2.03E-05	0.000774974	0.000607483
KEGG_ASCORBATE_AND_ALDARATE_METABOLISM	17	0.812537905	2.28295297	2.75E-05	0.000774974	0.000607483
KEGG_METABOLISM_OF_XENOBIOTICS_BY_CYTOCHROME_P450	54	0.578288585	2.09112091	2.71E-05	0.000774974	0.000607483
Low risk group						
KEGG_ASTHMA	20	−0.712928801	−1.470009905	0.032191311	0.1427422	0.111891982
KEGG_SYSTEMIC_LUPUS_ERYTHEMATOSUS	111	−0.619033995	−1.450026951	0.00039735	0.004309717	0.003378277

The table includes key parameters: Enriched pathways, names of KEGG pathways; Size, number of genes included in the pathway; Es, enrichment score; NES, normalized enrichment score; p value, raw p-value; p. adjust, adjusted p-value; and q value, false discovery rate-adjusted q-value. Positive Es and NES indicate pathways enriched in the high-risk group, while negative values indicate pathways enriched in the low-risk group. Statistical significance was defined as p. adjust <0.05 and q value <0.05.

### Association between the NECSO-related prognostic model and immune infiltration in LUAD

**FIGURE 11:**
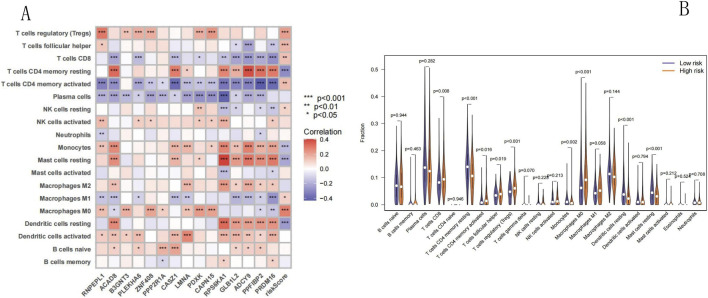
Tumor infiltrating immune cells analysis. **(A)** Heatmap: Correlation between infiltrating immune cells and risk score. **(B)** Vioplot: Differential immune cell abundance across risk groups. **(A)** Heatmap: This heatmap illustrates the correlation between the abundance of various tumor-infiltrating immune cells (rows) and the expression of NECSO-related genes or the risk score (columns). The color gradient (red for positive correlation, blue for negative correlation) and asterisk annotations (***p < 0.001, **p < 0.01, *p < 0.05) indicate the strength and statistical significance of these correlations. **(B)** Vioplot: This vioplot compares the abundance of tumor-infiltrating immune cells between low-risk (blue) and high-risk (orange) groups. Each plot displays the distribution of cell abundance, with p-values indicating statistical differences.

Using the CIBERSORT algorithm, we calculated the proportions of 22 immune cell types in the training set, combined them with the RS values, and visualized the immune cell correlations using a correlation heatmap (Figure 11A). The correlation heatmap revealed that 11 types of immune cells were highly correlated; among these, T regulatory cells (Treg), T follicular helper cells (Tfh), CD8^+^ T cells, activated memory CD4^+^ T cells, resting natural killer (NK) cells, M0 macrophages, and M1 macrophages exhibited a positive correlation; however, resting CD4^+^ memory T cells, monocytes, resting mast cells, and resting dendritic cells displayed a negative correlation. Subsequently, using the vioplot, the differences in immune cell expression in the low- and high-risk groups of the training set were compared (Figure 11B); the numbers of CD8^+^ T cells, Treg, monocytes, activated memory CD4^+^ T cells, and Tfh were elevated in the high-risk group compared to that in the low risk group (p < 0.05); however, the numbers of resting CD4^+^ memory T cells, M0 macrophages, resting dendritic cells, and resting mast cells were decreased in the high-risk group compared to that in the low risk group (p < 0.05). Collectively, these data imply that the NECSO-related LUAD prognostic model can be used to analyze immune cell infiltration in LUAD.

### Association between the NECSO-related prognostic model and TMB in LUAD

**FIGURE 12:**
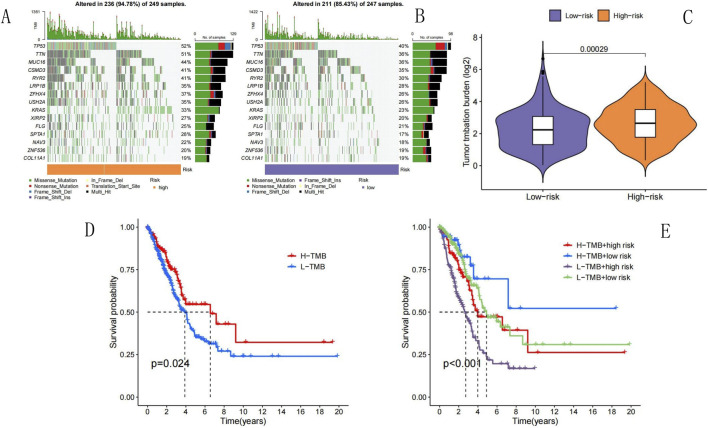
TMB analysis. **(A)** TMB in high-risk group. **(B)** TMB in low-risk group. **(C)** Differences in TMB between high-risk and low-risk groups. **(D)** Survival analysis by TMB status (high vs. Low). **(E)** TMB-risk survival analysis. **(A)** TMB in High-Risk Group: This oncoplot displays the top mutated genes in the high-risk group, with 236 out of 249 samples (94.78%) harboring genetic alterations. Genes like TP53 (52%), TTN (51%), and MUC16 (44%) are among the most frequently mutated. The color legend indicates mutation types, and the “Risk” bar at the bottom confirms all samples belong to the high-risk group. This high mutation burden suggests genomic instability in high-risk patients. **(B)** TMB in Low-Risk Group: This oncoplot shows mutations in the low-risk group, with 211 out of 247 samples (85.43%) altered. Top mutated genes include TP53 (40%), TTN (36%), and MUC16(36%). The “Risk” bar confirms low-risk status. **(C)** Differences in TMB Between High-Risk and Low-Risk Groups: This violin plot compares tumor mutation burden between groups. The high-risk group (orange) has a significantly higher TMB than the low-risk group (purple, p = 0.00029). **(D)** Survival Analysis by TMB Status (High vs. Low): The Kaplan-Meier curve shows survival differences between high TMB (H-TMB, red) and low TMB (L-TMB, blue) groups. **(E)** TMB-Risk Survival Analysis: This curve stratifies patients by both TMB and risk group.

We used the “maftools” package of R, version 4.4.0, to organize and analyze the mutation data in the TCGA dataset; a waterfall plot was used to present the mutation information of each gene in the sample. We comparatively ranked the top 15 mutated genes in the high- (Figure 12A) and low-risk (Figure 12B) groups; the findings showed that TP53 (52% vs. 40%, respectively), titin (TTN; 51% vs. 36%, respectively), mucin 16 (MUC16; 44% vs. 36%, respectively), CUB and sushi multiple domains 3 (CSMD3; 41% vs. 35%, respectively), low-density lipoprotein receptor-related protein 1B (LRP1B; 35% vs. 28%, respectively), zinc finger homeobox 4 (ZFHX4; 37% vs. 26%, respectively), Usher syndrome type 2A (USH2A; 35% vs. 26%, respectively), and Kirsten rat sarcoma viral oncogene homolog (KRAS; 33% vs. 23%, respectively) were the most prevalently mutated genes; the number of patients with TP53 and TTN mutations was significantly greater in the high-risk group than that in the low-risk group. Using a vioplot, we compared the differences in TMBs of the high- and low-risk groups in the training set; the high-risk group had a higher TMB level than that of the low-risk group (p = 0.00029) (Figure 12C). Comprehensive survival analysis performed using the Kaplan-Meier method (Figure 12D) revealed a notable difference in survival between patients with high and low TMB values (p = 0.024). In addition, Kaplan–Meier survival analysis was conducted in conjunction with examination of RS values (Figure 12E); the findings showed that the survival rates for patients with high TMB + low-risk scores were significantly higher than those for patients with high TMB + high-risk, low TMB + high-risk, and low TMB + low-risk scores (p < 0.001). These data show that the NECSO-related LUAD prognostic model was linked to the TMB in LUAD.

## Discussion

Lung cancer is the second most common malignant tumor worldwide and has the highest mortality rate (Sung et al., 2021; Zhou et al., 2021). In China, most lung cancer cases are diagnosed at advanced clinical stages, and the overall 5-year survival rate is low (He et al., 2021). Surgery combined with radiotherapy is the best treatment option for lung cancer. With the continuous advancement of research on lung cancer, remarkable progress has been made in the early screening, diagnosis, and treatment of LUAD. However, owing to its high heterogeneity and complex genetic inheritance and molecular mechanisms, LUAD is still one of the most fatal factors affecting human health.

In recent years, significant progress has been made in the study of programmed cell death. In addition to the classical apoptotic pathway, multiple novel cell death modalities such as necroptosis, scorch death, and iron death have been successively discovered. Unlike the previously known cell death modalities, NECSO is driven by a specific inward flow of sodium ions triggered by sustained activation of TRPM4 channels. This sodium overload forces Na^+^-K^+^ ATPases to overload, leading to rapid cellular energy depletion and mitochondrial dysfunction, ultimately causing necrotic cell death. NECSO is independent of the traditional apoptotic pathway and provides a new intervention to overcome apoptotic resistance in tumor cells. We can provide a new therapeutic option for the treatment of LUAD by means of NECSO. Therefore, in this study, we constructed a prognostic model for LUAD based on NECSO-related genes and analyzed immune cell infiltration and TMB using this model.

In this study, coexpression analysis of TRPM4 and TCGA LUAD RNA-seq data revealed the coexpression of 169 NECSO-related genes. GO and KEGG enrichment analysis of these 169 NECSO-related genes revealed that these NECSO-related genes were notably enriched in the regulation of protein secretion and membrane localization, oligosaccharide metabolism, and lysosome/vesicle membrane function and cadherin-binding and GTPase-regulatory activities. KEGG pathway analysis further showed that these genes were mainly involved in endocytosis and tight junction and actin cytoskeleton regulation. Therefore, we suggest that NECSO may be driven by a combination of membrane integrity disruption (such as lysosomal leakage or tight junction disassembly), ion transport dysregulation (such as sodium channel dysfunction), and cytoskeletal remodeling.

We used TCGA and GEO samples as the training and validation sets, respectively. Subsequently, we performed univariate Cox regression analysis of the training set and identified 20 genes significantly associated with sodium overload-related death. To further optimize the model and reduce the risk of overfitting, we analyzed the data using LASSO regression and finally obtained 15 NECSO-related genes that were closely associated with OS in patients with LUAD (RNPEPL1, B3GNT3, PLEKHA6, ZNF408, PPP2R1A, LMNA, PDXK, CAPN15, ACAD8, CASZ1, RPS6KA1, GLB1L2, ADCY9, PPFIBP2, and PRDM16). Among these, the expression levels of RBPEPL1, B3GNT3, PLEKHA6, ZNF408, PPP2R1A, LMNA, PDXK, and CAPN15 significantly increased with aggravation of the tumor condition, and the aggravation could be slowed down by inhibiting the expression of these genes. In contrast, upregulation of the expression of ACAD8, CASZ1, RPS6KA1, GLB1L2, ADCY9, PPFIBP2, and PRDM16 contributed to disease retardation. However, the specific molecular mechanisms underlying LUAD have not yet been elucidated and require further studies.

We constructed a prognostic model based on 15 NECSO-related genes that could predict OS in patients with LUAD. Univariate and multifactorial Cox regression analyses revealed that the model was an independent prognostic indicator for LUAD. This prognostic model accurately categorized patients into low- and high-risk groups. The low-risk group showed a better prognosis than that of the high-risk group in both the training and validation sets. Furthermore, we evaluated the accuracy of this prognostic model in predicting prognosis by constructing an ROC curve for the training set. The findings revealed that the AUC values for 1, 3, and 5 years were 0.717, 0.703, and 0.643, respectively; the results indicated that the model predicted survival rates after 1 and 3 years of diagnosis with high accuracy; however, the predictive ability for survival after 5 years was lesser than that for survival after 1 and 3 years of diagnosis. Therefore, improvement of the prognostic model in subsequent studies and expansion of the sample data to enhance the predictive accuracy of the model are necessary.

A major difficulty in the treatment of patients with LUAD is that the tumor is highly heterogeneous and existing immunotherapeutic agents are not suitable for all patients with LUAD. Therefore, identification of novel immunotherapeutic targets and prognostic indicators is important. In our study, the mutation frequencies of key genes such as TP53 (52% vs. 40%, respectively) and TTN (51% vs. 36%, respectively) were significantly higher in the high-risk group than those in the low-risk group (p < 0.05); this result is in line with recent findings that TP53 mutations promote tumor genomic instability and lead to a poor prognosis (Olivier et al., 2010). Moreover, the high-risk group exhibited higher TMB levels than those of the low-risk group (p = 0.00029); this finding is consistent with the theory that the accumulation of mutations during tumor evolution leads to increased malignancy (Chan et al., 2019). Furthermore, Kaplan–Meier survival analysis revealed that the survival rate of patients with high TMBs and low RS values was significantly higher than that of the other subgroups (p < 0.001); this finding not only validated the reliability of the prognostic model but also suggested that NECSO-related genes might be involved in the progression of LUAD by regulating the tumor mutation process. The mechanism by which NECSO-related genes improve the prognosis of LUAD by affecting TMB should be further analyzed in subsequent studies.

Furthermore, tumor-infiltrating immune cells may be of prognostic significance in cancer treatment. One core feature of tumor immune microenvironment disorder is increased immunosuppressive cell infiltration and impaired host immune function, which is proven closely linked to poor prognosis and suboptimal treatment response in LUAD patients (Ke et al., 2025; Zhang et al., 2025). In this study, the CIBERSORT algorithm was used to analyze the relationship between the NECSO-related prognostic model and tumor immune microenvironment, revealing a significant correlation between the RS and infiltration of specific immune cell subpopulations. The findings showed that the high-risk group exhibited unique immune characteristics: 1) a significant increase in the numbers of immunosuppressive cells such as Treg, depleted T cells such as CD8^+^ T cells, and activated memory CD4^+^ T cells (p < 0.05), which is highly consistent with recent findings on the immune escape mechanism of tumors (Zhao et al., 2021); 2) an imbalance in the ratio of pro-inflammatory cells such as M1 macrophages to anti-inflammatory cells such as M0 macrophages; and 3) a significant reduction in the numbers of antigen-presenting cells such as resting dendritic cells. These findings demonstrated that NECSO-related genes may regulate immune cell functions in the tumor microenvironment, especially the activation and effector functions of T cells, by affecting the network of interactions between immune checkpoints and ion channels. Notably, the increase in Tfh numbers may be associated with the modulation of B-cell function (Garaud et al., 2022), implying that sodium ion disruption may affect the humoral immune response; however, whether NECSO-related genes directly regulate immune checkpoint expression remains unclear. Therefore, exploration of the regulatory mechanisms of key genes (such as the risk gene RNPEPL1) in immune cell function is required in future studies.

Identifying additional molecular pathways associated with LUAD may facilitate the discovery of new therapeutic targets. In this study, we used GSEA to screen signaling pathways implicated using the prognostic model of NECSO-related LUAD; the results showed that the high-risk group was significantly enriched in metabolism-related (such as ascorbic and aldosteric acid metabolism and cytochrome P450-mediated metabolism of exogenous substances) and proliferation-related (such as DNA replication) pathways; these findings suggested that the tumor cells progressed to malignancy via enhancement of metabolic adaptations and proliferative capacity; however, the low-risk group was enriched in immune-related pathways (such as asthma and systemic lupus erythematosus), demonstrating that autoimmune-like responses may unexpectedly enhance antitumor immune surveillance (Subramanian et al., 2005; Liberzon et al., 2011). These findings imply that NECSO-related genes may regulate LUAD through metabolic–immune interaction networks, which may enable development of targeted therapeutic strategies in the future based on the theoretical basis of RS stratification.

In clinical practice, tumor staging is defined by the tumor size, lymph node, and metastasis (TNM) approach (Chansky et al., 2017), which is usually used to assess the prognosis of patients with tumors. Although TNM provides an important indicator of disease severity by objectively assessing the anatomical characteristics of the tumor, it does not consider factors such as sex and age that may affect prognosis. The nomogram constructed in this study conformed to the guidelines of the American Joint Committee on Cancer (AJCC) eighth edition of the cancer staging system (stages I–IV) (Amin et al., 2017); this system also incorporates several aspects such as the sex parameter (May et al., 2023), age factor (Gammelgaard et al., 2024), and NECSO-related gene RS for assessment of prognosis compared to the use of the cancer staging system alone. The nomogram quickly obtains an individualized survival prediction for a patient by converting each predictor into a standardized score through an intuitive graph; the predictions can be ascertained by clinicians by simply calculating the total score and drawing a vertical line on the total score axis of the outcome axis. The calibration curves showed that the predicted results were highly consistent with the observed values and had high predictive accuracy. In contrast to the limitations of previous studies, including the broad definition of predicted outcomes, the lack of accurate subgroup-specific prediction, the single dimension of prognostic assessment without comprehensive multi-endpoint evaluation, and the restricted predictive scenarios with a narrow applicable population that compromise their clinical utility, the predictive model established for lung adenocarcinoma (LUAD) patients in this study not only enables precise risk stratification of patients but also undergoes comprehensive validation based on multiple core prognostic outcomes including overall survival (OS) and time to disease progression. This markedly enhances the comprehensiveness and reliability of model prediction. Furthermore, the model is applicable to a broad LUAD population, which effectively overcomes the bottleneck of limited applicable scenarios in similar studies and thus provides a more practically valuable reference for the clinical implementation of individualized diagnosis and treatment for LUAD patients (Mallardo et al., 2025; Mallardo et al., 2023; Mallardo et al., 2024). The findings provide clinicians with a reliable method for individualized prognosis assessment, helping to guide the development of targeted therapeutic regimens. NECSO is a novel mode of cell death that may provide a new approach for the treatment of LUAD. Our study contributes to a better understanding of NECSO-related LUAD prognostic models and provides insights into the development of new therapeutic approaches.

## Conclusion

In this study, expression data and the corresponding clinical information on patients with LUAD were obtained from the TCGA and GEO databases and used as the training and validation sets, respectively. Coexpression analysis of the training set and NECSO gene TRPM4 was performed; 169 NECSO-related genes were filtered out, which were subjected to GO and KEGG enrichment analyses. Using univariate Cox regression analysis, we obtained 20 significantly expressed NECSO-related genes; to reduce the risk of overfitting, we performed LASSO regression analysis to obtain 15 NECSO-related genes and used them to construct prognostic models. Meanwhile, we integrated the results of independent prognostic analyses and constructed a nomogram. Finally, based on the prognostic model, we stratified patients with LUAD into high- and low-risk groups. Moreover, we performed GSEA and TMB and immune infiltration analysis of the two groups, which provided new insights into the clinical development of personalized treatment. The main conclusions are as follows:

1. On the basis of bioinformatic analysis, we constructed a prognostic model for NECSO-related LUAD, including eight risk genes (RBPEPL1, B3GNT3, PLEKHA6, ZNF408, PPP2R1A, LMNA, PDXK, and CAPN15) and seven protective genes (ACAD8, CASZ1, RPS6KA1, GLB1L2, ADCY9, PPFIBP2, and PRDM16). The model was validated to accurately predict the prognosis of patients with LUAD. Information on clinical factors was integrated into this model and a column-line diagram was established to predict the survival outcomes of patients with LUAD; the model showed good predictability.
2. Multiomics analysis revealed characteristic molecular alterations in high-risk patients with LUAD; high-risk patients had a significantly higher frequency of mutations in the TP53 and TTN genes and a significantly increased tumor mutational load compared to those in low-risk patients. Analysis of the immune microenvironment revealed a high number of immunosuppressive cells and a strikingly diminished antigen presentation function in the high-risk patients. In terms of metabolic characteristics, the ascorbic acid metabolic pathway and the DNA replication pathway showed an abnormally active state in these patients.

In this study, the pathogenesis of LUAD was investigated in combination with NECSO. The study provides new molecular markers and potential therapeutic targets for precise prognostic prediction and individualized treatment and has clinical significance. Future studies should explore the specific regulatory mechanisms of sodium overload-induced death in LUAD and develop targeted therapeutic programs.

## Data Availability

The datasets supporting the conclusions of this article are included within the article. The data analyzed in this study were obtained from the TCGA database (https://portal.gdc.cancer.gov/) and the GEO database (http://www.ncbi.nlm.nih.gov/geo) with the accession numbers GSE68465 and GPL96. The analysis software is included the R language (version 4.4.0, https://cran.r-project.org/bin/windows/base/old/4.4.0/) and Perl (version 5.30.0, https://www.perl.org/).

## Glossary

ACAD8: acyl-CoA dehydrogenase 8
ADCY9: adenylate cyclase 9
B3GNT3: beta-1,3-N-acetylglucosaminyltransferase 3
BP: biological process
CAPN15: calpain 15
CASZ1: castor zinc finger 1
CC: cellular component
CSMD3: CUB and sushi multiple domains 3
GLB1L2: galactosidase beta 1 like 2
KRAS: Kirsten rat sarcoma viral oncogene homolog
LASSO: least absolute shrinkage and selection operator
LMNA: lamin A/C
LRP1B: low-density lipoprotein receptor-related protein 1B
LUAD: lung adenocarcinoma
MF: molecular function
MUC16: mucin 16
NECSO: necrotic cell death triggered by sodium overload
NSCLC: non-small cell lung cancer
OS: overall survival
PDXK: pyridoxal kinase
PLEKHA6: pleckstrin homology domain-containing family A member 6
PPFIBP2: protein tyrosine phosphatase receptor type F interacting protein-binding protein 2
PPP2R1A: protein phosphatase 2A
PRDM16: PR domain-containing protein 16
RNPEPL1: arginyl aminopeptidase like 1
RPS6KA1: ribosomal protein S6 kinase A1
RS: risk score
TMB: tumor mutational burden
TRPM4: transient receptor potential melastatin 4
TTN: titin
USH2A: Usher syndrome type 2A
ZFHX4: zinc finger homeobox 4
ZNF408: zinc finger protein 408
